# Effect of Mycotoxins in Silage on Biogas Production

**DOI:** 10.3390/bioengineering10121387

**Published:** 2023-12-02

**Authors:** Antonín Kintl, Tomáš Vítěz, Igor Huňady, Julie Sobotková, Tereza Hammerschmiedt, Monika Vítězová, Martin Brtnický, Jiří Holátko, Jakub Elbl

**Affiliations:** 1 Agricultural Research, Ltd., Zahradní 1, 664 41 Troubsko, Czech Republic; kintl@vupt.cz (A.K.); hunady@vupt.cz (I.H.); sobotkova@vupt.cz (J.S.); 2 Department of Agricultural, Food and Environmental Engineering, Faculty of AgriSciences, Mendel University in Brno, Zemědělská 1, 613 00 Brno, Czech Republic; 3 Department of Experimental Biology, Section of Microbiology, Faculty of Science, Masaryk University, Kamenice 753/5, 625 00 Brno, Czech Republic; vitezova@sci.muni.cz; 4 Department of Agrochemistry, Soil Science, Microbiology and Plant Nutrition, Mendel University in Brno, Zemědělská 1, 613 00 Brno, Czech Republic; tereza.hammerschmiedt@mendelu.cz (T.H.); martin.brtnicky@mendelu.cz (M.B.); jiri.holatko@mendelu.cz (J.H.); 5 Agrovyzkum Rapotin, Ltd., Vyzkumniku 267, 788 13 Rapotin, Czech Republic; 6 Department of Agrosystems and Bioclimatology, Faculty of AgriSciences, Mendel University in Brno, Zemědělská 1, 613 00 Brno, Czech Republic

**Keywords:** anaerobic biogas, methane, mycotoxins, maize silage, anaerobic digestion, digestate

## Abstract

Mycotoxins can pose a threat to biogas production as they can contaminate the feedstock used in biogas production, such as agricultural crops and other organic materials. This research study evaluated the contents of deoxynivalenol (DON), zearalenone (ZEA), fumonisin (FUM), and aflatoxin (AFL) mycotoxins in maize silage prior to it being processed in a biogas plant and in digestate produced at the end of the anaerobic digestion (AD) process. In the experiment, three samples of silage were collected from one silage warehouse: Variant 1 = low contamination, Variant 2 = medium contamination, and Variant 3 = heavy contamination, which were subjected to investigation. A significantly reduced biogas production was recorded that was proportional to the increasing contamination with molds, which was primarily due to the AD of silage caused by technologically erroneous silage treatment. The AD was connected with changes in silage composition expressed by the values of VS content, sugar content, lactic acid content, acetic acid content, and the ratio of lactic acid content to acetic acid content. The production of biogas and methane decreased with the increasing contents of NDF, ADF, CF, and lignin. The only exception was Variant 2, in which the content of ADF, CF, and lignin was lower (by 8–11%) than that in Variant 1, and only the content of NDF was higher (by 9%) than that in Variant 1. A secondary factor that also correlated with changes in the composition of the substrate was the development of undesirable organisms, which further contributed to its degradation and to the production of mycotoxins. It was also demonstrated in this study that during the AD process, the tested mycotoxins were degraded, and their content was reduced by 27–100%. Only the variant with low mold contamination showed a DON concentration increase of 27.8%.

## 1. Introduction

Cereals (wheat, rice, maize, etc.) are the most important agricultural crops worldwide, the main reason being their relatively high nutritional value and ease of growth [1]. According to the FAO [2], the annual global growing area of maize and wheat in 2020 was ca. 202 and 219 million hectares, respectively, with maize being widely used as a food or fodder crop. The production of silage is a widely used practice to retain the nutritional value of farm animal feeds made of cereal biomass, most frequently by means of spontaneous lactic fermentation in anaerobic conditions [3].

Nevertheless, depending on growing conditions, cereals can be infested by multiple strains of toxicogenic fungi, with the majority of them producing more than one type of mycotoxin [1]. Mycotoxins are secondary metabolites of fungi with diverse chemical structures that contaminate many of the most frequently consumed food and feed products worldwide [4,5]. There are two ways for cereals to be contaminated by mycotoxins: (i) fungi growing as pathogens on plants or (ii) saprophytically on stored crops [6]. In the past, mycotoxins were recorded particularly in wheat and maize grains, which exhibited the highest concentrations of fumonisin (FB), deoxynivalenol (DON), aflatoxin (AF), and zearalenone (ZEN) [1].

The development of mycotoxins depends on a range of conditions during the pre-harvest and post-harvest handling of agricultural products that can be divided according to Adegoke and Letum [7] and Magan et al. [8]: (A) internal factors: moisture content, water activity, substrate type, plant type, and composition of nutrients; (B) external factors: climate, temperature, oxygen level; (C) factors of processing: drying, mixing, addition of preservatives, grain handling; and (D) implicit factors: interaction with insects, strains of fungi, microbiological ecosystem.

Adegoke and Letum [7] also include higher temperatures, abundant rains during harvest (biomass moisture content), stand density, mechanical harvesting, vegetation stage, drought, variety, soil structure, and temperatures below freezing as factors participating in the spread of molds and the development of mycotoxins. Warmer climatic conditions promote a more frequent occurrence of aflatoxins and fumonisins. In contrast, colder regions with high humidity support the development of ochratoxins, zearalenone, deoxynivalenol (DON), T-2 toxin, and diacetoxyscirpenol (DAS) [9]. The three most frequently occurring toxicogenic fungi in Europe are *Aspergillus*, *Penicillium*, and *Fusarium* [10].

In farm animals, the consumption of feeds contaminated by mycotoxins may have unfavorable consequences such as reduced feed intake, refusal of feed, poor feed conversion, reduced increment in body weight, increased occurrence of diseases (due to suppressed immunity), and impaired capacity of reproduction [11,12], which lead to economic losses [13,14]. Many batches of cereals cannot be used for food, feed, or energy because of their high contamination by mycotoxins. In recent years, increasing attention has been given to renewable sources of energy that can replace fossil fuels. The process of anaerobic digestion (AD), whose goal is the production of biogas from diverse organic waste materials and suitable energy crops, appears to be promising [15,16]. In addition to biogas, a side product of the AD process is digestate, which can have high agronomic value as fertilizer thanks to the high content of mineral nitrogen and relatively stable organic matter [17,18]. Digestate was demonstrated to have positive effects in agriculture on the growth and production of crops if applied to post-harvest residues or in combination with readily degradable organic matter [19] as compared with other kinds of organic fertilizers [20]. Processes taking place during the AD of the organic feedstock enhance the properties of the biomass source; e.g., it improves the availability of N in digestate and P in plants [21]. Thanks to these properties, fertilization based on digestate can be as effective as conventional organic supplements such as farmyard manure or compost [22].

Thus, AD may represent an interesting approach to biogas production from organic waste materials (such as silage) contaminated by mycotoxins. However, when using material contaminated by mycotoxins as a feedstock for biogas production, one has to consider the possible negative influences on the process of AD, biogas yield, and digestate quality [1,22]. These aspects were dealt with in several studies that revealed reduced contents of several mycotoxins in mesophilic and thermophilic conditions, both in batch tests and in semi-continual reactors [23,24,25]. The main problem with using silages highly contaminated with mycotoxins is their influence on microbial diversity in the fermenters of biogas plants. The process of AD itself can reduce the content of mycotoxins by more than 50% [1] because some mycotoxins are susceptible to biological degradation [26]. However, the problem during the AD of silage contaminated by mycotoxins is the decreased production of biogas [27]. The reason is simple: increased concentrations of mycotoxins impair the diversity of the microbial community [1], increase the accumulation of organic acids, and decrease the pH value in the fermenter [28]. All these effects then result in AD inhibition and, hence, the reduced production of biogas [1,27,28]. In the process of AD, individual types of mycotoxins exhibit different potentials for biodegradation [27,28]. The mycotoxins with the greatest potential for biodegradation are AF B_1_, FM, and DON. It was found that the process of biodegradation through AD can reduce the concentration of these mycotoxins by more than 50% [1,27,28]. It is also necessary to realize that silage AD gives rise not only to biogas but also to fermentation residues in the form of digestate, which can be contaminated by mycotoxins too [28]. Additionally, when digestate is used as fertilizer, it is necessary to investigate its influence on the quality of food and feed products not only in terms of nutritional value [29], but also in terms of soil properties [30,31], as well as the possible contamination of the environment with mycotoxins [32].

This study presents a novel combined evaluation of the content of mycotoxins in the contaminated silage, their effect on biogas and methane production, and the degradation of mycotoxins during the AD process (monitored as the residual content of mycotoxins in the digestate). This study has the following objectives: (1) to determine the degree to which the presence of mycotoxins in contaminated silage affects the production of biogas or methane and (2) to determine whether degradation of tested mycotoxins takes place in the process of silage AD. Furthermore, the hypotheses of this study are as follows: H_0_: Silage contamination by mycotoxins does not affect biogas and methane production during anaerobic digestion. H_1_: Degradation of tested mycotoxins does not occur in the process of silage AD.

## 2. Materials and Methods

### 2.1. Organization of the Experiment—Collection of Contaminated Samples

The experiment was based on testing contaminated silage under laboratory conditions with regard to biogas production and analyzing some qualitative indicators. A silage pit with visible mold infestation was selected for the experiment. 

**Figure 1:**
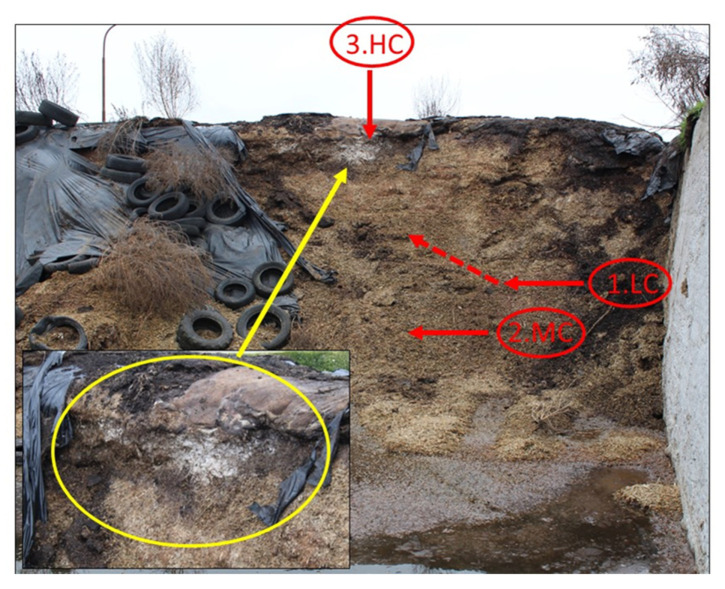
Localization of experimental variants characterized by the sampling layers and the depth of silage in the silage pit, determined as follows: Variant 1 = low contamination (LC)—sampled at a depth of 50 cm; Variant 2 = medium contamination (MC); Variant 3 = heavy contamination (HC).

Three variants of maize silage were sampled from the silage pit (Figure 1): Variant 1 = low contamination (LC)—visibly non-infected sample; Variant 2 = medium contamination (MC); Variant 3 = heavy contamination (HC). The variants were of different quality in terms of visible mold infestation (Figure 1), with three replicates each, i.e., a total of nine samples.

### 2.2. Silage Characteristics

The selected qualitative indicators (N-substances, crude fiber, acid detergent fibers, etc.) were analyzed in the collected samples of contaminated silage to determine the effects of mycotoxin contamination on silage quality. N-substances were determined by the Kjeldahl method according to ISO 20483:2013 [33] using the KjeltecTM 2300 analyzer (FOSS Analytical, Hillerød, Denmark), and the protein content was calculated from the N-substance value by multiplying the N-substance value by the empirical factor 6.25. The fat content was determined gravimetrically using the water-cooled Soxhlet [34] extractor BEHR 6 (Behr Labor-Technik GmbH, Düsseldorf, Germany) by direct sample extraction with diethyl ether. The crude fiber (CF) content was determined using two-stage hydrolysis with sulfuric acid and potassium hydroxide. The ash content was determined according to ISO 6865:2000 [35]. The acid detergent fiber (ADF) content was determined using a solution of concentrated sulfuric acid and cetyltrimethylammonium bromide (CTAB). The acid detergent lignin (ADL) was determined according to ISO 13906:2008 [36]. Neutral detergent fiber (NDF) was determined using a solution of sodium lauryl sulfate and ethylenediaminetetraacetic acid according to ISO 16472:2006 [37]. The following parameters were determined during silage analysis: dry matter, VS, ash, pH, aqueous solution, lactic acid, acetic acid, propionic acid, and butyric acid. The content of dry matter (=total solids, TS/DM) and volatile solids in the samples was determined gravimetrically by drying in the electric furnace LMH 07/12 (LAC, Židlochovice, Czech Republic) at 105 °C to constant weight and by annealing the dried samples at 550 °C to constant weight in accordance with the standards ČSN EN 15934 [38] and ČSN EN 15935 [39]. All the parameters mentioned, including the description of their determination, have already been given in the work of Hunady et al. [40] and Kintl et al. [41].

The starch content of the silage was determined using the polarimetric method (Polamat S, Carl Zeiss Jena GmbH, Jena, Germany) in accordance with ISO 6493:2000 [42]. The fat content was determined gravimetrically using the water-cooled Soxhlet extractor by direct sample extraction with petroleum ether [43]. The content of sugars (reducing saccharides) was determined using the Luf–Schoorl method [44,45,46]. The pH of the H_2_O leachate was determined according to ČSN 46 7092-42 [47]. Lactic acid, acetic acid, propionic acid, and butyric acid were determined according to Stringer [48]—using the IONOSEP 2001 capillary isotachophoresis instrument (RECMAN—laboratory technique, Ltd., Ostrava, Czech Republic).

### 2.3. Digestion Batch Tests

**Figure 2:**
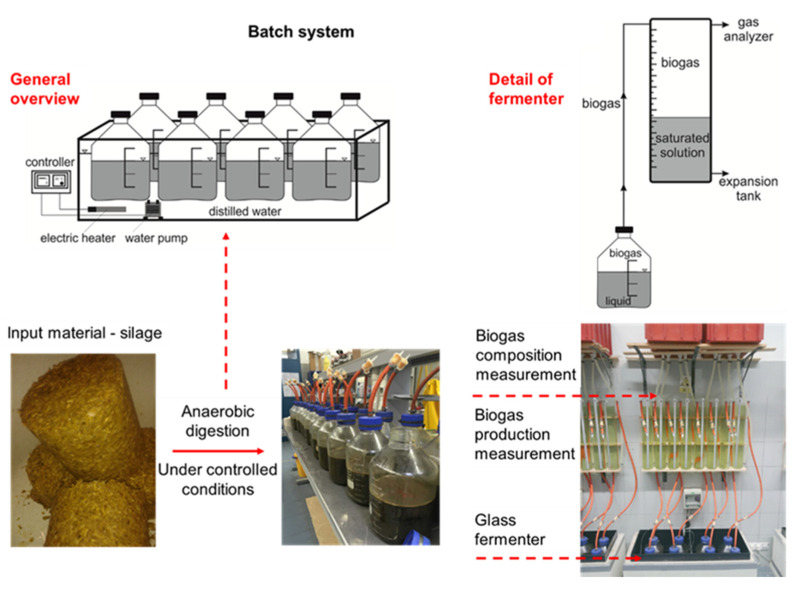
Fermentation test design (Kintl et al. [50]).

The digestion batch tests were performed according to Kintl et al. [41], using systems for batch tests (Figure 2). The inoculum was digestate from the biogas plant in Čejč, Czech Republic, processing maize silage and slurry, with the following parameters: mesophilic temperature conditions, 38 °C; dry matter, 3.43 ± 0.06%; volatile solids, 68.54 ± 0.11%; pH, 7.0; FOS, 740 mg/L; TAC, 2200 mg/L; NH_4_^+^, 590 mg/L. The biogas composition was analyzed using a Dräeger X-am 5600 biogas analyzer (Drägerwerk AG & Co. KGaA, Lübeck, Germany), as described in the work of Kintl et al. [49] and Kintl et al. [50]. The volume of biogas generated was converted to standard temperature and pressure (273.15 K and 1 bar). The process parameters were as follows: initial organic loading rate = 4.5 g_vs_ introduced substrate/L; retention time = 21 days; temperature during the test = 42 °C ± 0.1 °C. The silage parameters can be found in Table 1 in the Results section.

### 2.4. Analysis of Mycotoxins

Samples of silage and digestate were dried at 60 °C and then analyzed for the content of mycotoxins deoxynivalenol (DON), aflatoxin (AFL), zearalenone (ZEA), and fumonisin (FUM) using the enzymatic immunosorbent assay (ELISA) according to Skládanka et al. [51]. The ELISA (MyBioSource, San Diego, CA, USA) is a competitive direct enzyme test for the qualitative analysis of plant biomass focused on the content of mycotoxins. The concentrations of individual mycotoxins were expressed in micrograms per kg of silage or digestate [52].

### 2.5. Statistical Analysis—Data Treatment

All experimental parameters were measured in three replicates. The data were processed in the Statistica 14 program (TIBCO Software, Inc., Palo Alto, SF, USA). The procedures used to analyze the data included exploratory data analysis (EDA), one-way analysis of variance (ANOVA), and Tukey’s HSD post hoc test. The data were standardized, and the relationships between the values of the measured parameters were analyzed using correlation analysis, factor analysis (FA), principal component analysis (PCA), and cluster analysis (CA). All analyses were performed at a significance level of *p* < 0.05.

## 3. Results and Discussion

### 3.1. Qualitative Parameters of Silage

The quality of the individual silage variants was assessed by determining the following parameters: volatile solids content (VS), dry matter (DM), neutral detergent fiber (NDF), acid detergent fiber (ADF), crude fiber (CF), lignin, proteins, starch, sugars, lactic acid (LA), acetic acid (AA), titratable acidity (TA), and pH. The measured parameters are divided into three subsections that characterize the content or the state of selected indicators: Section 3.1.1, DM, pH, VS, proteins, lipids, sugar; Section 3.1.2, Starch, NDF, ADF, CF, lignin; Section 3.1.3, LA, AA, LA/AA, TA, propionic acid, butyric acid. In general, measurement data show that contamination of the silages with molds leads to a deterioration in their qualitative indicators. The development of the measured indicators confirms the importance of adhering to the correct ensiling process [53]: (a) appropriate harvest date (dry matter up to 35%); (b) quality of the chopped material—a length of 15–25 mm is sufficient at the optimal stage of maize maturity; (c) ensiling process—oxygen must be displaced from the harvested matter (shreddings), and then further access of oxygen into the silage must be prevented. An anaerobic environment has to be created in which no atmospheric oxygen is supplied. Only then can the respiration of cells stop and the fermentation of the lactic acid bacteria begin. A necessary factor that influences the quality of the resulting silage is also the supplementation of silage additives, most commonly lactic acid bacteria.

#### 3.1.1. DM, pH, VS, Proteins, Lipids, and Sugar

The DM content in the silage ranged from 34.3% (MC variant) to 47.9% (HC variant). Differences between the variants were statistically significant (Table 1). According to Wilkinson [54], the ideal DM content in silage is 30–35%, with excessive DM content generally associated with susceptibility to molds. This indicates that all variants in our experiment with the exception of MC had a higher dry matter content than the value described as optimal by Wilkinson [54], creating the conditions for the development of molds (particularly in HC).

The pH value in the aqueous solution (Table 1) increased significantly with increasing optical contamination from 4.22 (LC) and reached 6.10 in the HC variant. Kung et al. [55] state that a normal pH value for silage with a DM content of 30–40% is 3.7–4.0. The pH values of our test variants were higher, and significantly higher again in the HC variant. As the pH value is a physical factor that ensures the preservation of the silage, pH values above 6 indicate the probable development of molds and are evidence of poor silage management [56].

The main cause of the drop in silage pH is the production of lactic acid (LA), a typical fermentation product during the ensiling of maize, the amount of which is significantly higher than the amount of other organic acids (acetic, propionic, and butyric acids) normally detected during ensiling [57]. This is also confirmed by the results of our study, which showed a very strong negative correlation (R = −0.94) between the pH value and the concentration of LA in the silage (Table A1).

Silage-degrading microorganisms are more effectively inhibited at lower pH values than at higher ones. In the past, silages with a pH value of more than 4.2 were considered of poor quality [58]. Currently, silages with a high DM content are produced on a large scale and are stable even at relatively high pH values. Although pH remains one of the most important indicators of good fermentation in low-DM silages, it is not a reliable indicator in high-DM silage [59]. This research also shows that DM content is very strongly correlated with pH (R = 0.95) and that the values of both parameters increase with increasing contamination of silage.

The VS content ranged from 92.05%_DM_ (MC) to 94.38%_DM_ (LC) and was significantly highest in the low contamination (LC) variant (Table 1). According to Heuzé et al. [60], the average VS content in maize silages with DM content of 35–40% ranges from 94.8 to 97.8%_DM_. Wilkinson [54] claims that an optimum VS content should be above 92%_DM_.

The content of proteins, lipids, and sugars showed significant differences between the variants. The highest protein content was found in the HC variant, and the highest lipid and sugar contents were found in the LC variant (Table 1). Heuzé et al. [60] state that the average content of proteins and lipids in maize silage with 35–40% dry matter is approx. 6.8%_DM_ and 3.0%_DM_, respectively. Therefore, we assume that the organic substances were degraded during the ensiling process and subsequently contaminated by molds. As microorganisms, molds first utilize readily available organic substances (sugars) and only then utilize the more complex forms such as lipids [61]. Although this explains the reduced content of lipids and sugars, it does not explain the slightly increased content of proteins, which could have been caused by protein-based substances produced by molds [62]. Kung et al. [55] found that clostridial silages, for example, are often characterized by higher than normal pH and higher than normal soluble protein concentrations. The mycelial biomass of various fungi can be used as a rich source of mycoproteins [63], and the production of microbial proteins by filamentous fungi is therefore a prospective bioprocess in the food and feed industry [64]. In some fungal groups, the mycelium is able to convert the otherwise difficult-to-process lignocellulosic substrates into proteins and can therefore be used as a sustainable source [65].

#### 3.1.2. Starch, NDF, ADF, CF, and Lignin

The experimental variants did not differ statistically in their starch content. However, the fiber (NDF, ADF, CF, Table 2) and lignin contents were significantly highest in the HC variant. Heuzé et al. [60] claim that the average starch content in maize silage with 35–40% dry matter is approx. 31.6%_DM_. Based on a meta-analysis, García-Chávez et al. [66] give an average value for the starch content of 23.3%_DM_.

#### 3.1.3. LA, AA, LA/AA, TA, Propionic Acid, Butyric acid

The LA content ranged from 0.65%_DM_ (HC) to 1.44%_DM_ (LC), and the difference between LC and MC on the one hand and HC on the other was statistically significant (Table 3). Kung and Shaver [57] claim that the usual values of LA concentration in maize silages with 30–40% dry matter are between 4 and 7%_DM_.

Similarly, the AA content decreased with increasing contamination, although the differences between the variants were statistically significant. According to Wilkinson [54], the optimum AA content in maize silage is 2–3%_DM_. In our experiment, the concentrations of both LA and AA were very low in all variants (Table 3) and were below the values described as optimal.

According to Kung et al. [55], lactic acid is one of the most important substances indicating the course of fermentation in silages with a low dry matter content. During the ensiling process, the LA produced by the lactic acid bacteria usually occurs in the highest concentration and is most strongly involved in lowering the pH value during fermentation, as it is about 10 to 12 times stronger than all other major acids [55]. However, in silages with a high DM content, the LA content does not always indicate successful fermentation [59]. Fermentation during which LA is produced leads to the lowest losses of dry matter and energy during storage [57]. The LA/AA ratio is commonly used as an indicator of the quality of fermentation. During optimal silage fermentation, the ratio of these acids usually ranges from approximately 2.5 to 3.0 [55]. An LA/AA ratio below 1 is usually an indication of abnormal fermentation. Low concentrations of AA may not be sufficient to inhibit lactate-assimilating yeast [55,57].

In our experiment, the LA/AA ratio ranged from 0.94 (MC variant) to 1.99 (HC variant) and was statistically significantly highest in the HC variant compared to the other two variants (Table 3). This shows that the values of all three variants were below the interval considered to be optimal. One reason for this was the very low content of LA, which was far below the usual values in maize silage [57]. The values of all parameters thus clearly indicate an increase in aerobic degradation, which correlates with the degree of silage contamination by molds. The aerobic phase can change the chemical composition of the silage after the silo has been opened. Increased pH (>6.0) caused by the growth of yeasts responsible for silage degradation leads to active growth of toxicogenic fungi during the feeding phase, especially in poorly treated silages [56]. The presence of propionic acid and butyric acid was below the detection limit of 10 mol/L.

### 3.2. Content of Mycotoxins in Silage

The levels of mycotoxins (DON, ZEA, FUM, AFL) found in maize silage in this study were on average lower than the values reported in the available literature, and the degree of contamination was not able to be distinguished [67,68,69,70].

#### 3.2.1. DON

**Figure 3:**
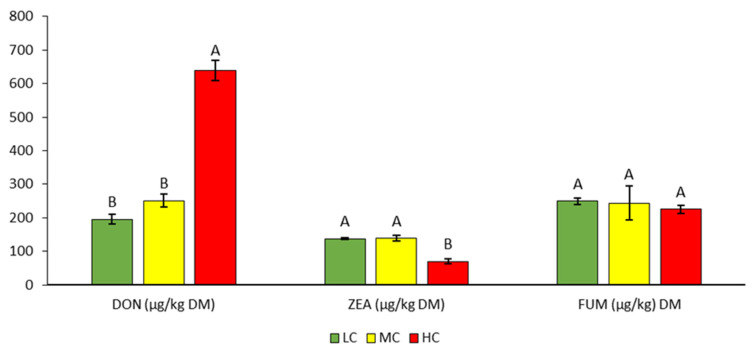
Contents of DON, ZEA, and FUM mycotoxins in silage. Different letters indicate significant differences (*p* < 0.05) between variants (LC, MC, HC) in the specific parameter (content of individual mycotoxin).

The average DON concentration in all samples was 361.7 µg/kg_DM_ and ranged from 180 to 670 µg/kg_DM_ (Table 4, Figure 3). The DON content increased with increasing contamination of the samples and was statistically significantly higher in the HC variant (638.3 µg/kg_DM_) than in the LC and MC variants (195.7 and 251.0 µg/kg_DM_).

Based on the previous studies, it can be stated that DON is one of the most frequently detected mycotoxins in silage, and its concentrations can be very high [71,72]. The average values given in the literature for the DON content in maize range from 280 [72] to 3142 µg/kg_DM_ [69]. A worldwide study of samples of feed for livestock, which lasted 3 years, showed that DON is a common threat to livestock with an occurrence of 59% and an average contamination of 1104 μg/kg [73]. In the Netherlands, a new study was carried out on the presence of mycotoxins in silages. It was found that the major sources of DON in dairy cattle feed are maize and wheat silages with average concentrations of 854 and 621 μg/kg and maximum concentrations of 3142 and 1165 μg/kg, respectively [69]. Another recent multi-year study of mycotoxins in Poland [70] revealed the presence of DON in 86% of 143 samples of maize silage with average and maximum concentrations of 223 and 7860 μg/kg, respectively.

The DON content we found in the most contaminated HC variant (638.3 µg/kg_DM_) was therefore rather below average compared to the average values mentioned in the above studies.

#### 3.2.2. ZEA

The average content of ZEA was 115.3 µg/kg_DM_ and ranged from 63 to 147 µg/kg_DM_ (Table 4). Compared to LC and MC, the statistically significantly lowest ZEA content was found in the HC variant (Figure 3).

The values mentioned in the literature range on average from 66 [68] to 432 µg/kg_DM_ [74]. The production of ZEA is supported by conditions with high humidity and alternating low (11–14 °C) and mild (27 °C) temperatures [75]. Whitlow and Hagler [76] reported an average ZEA concentration of 525 μg/kg_DM_ and an occurrence of 30% in 461 samples of maize silage in the USA. In the Netherlands, Driehuis et al. [69] detected ZEA in 50% of 140 maize silage samples, with an average concentration of 146 μg/kg_DM_. According to Storm et al. [68], zearalenone was the most frequently detected mycotoxin in a study evaluating the exposure of Danish cattle to mycotoxins from maize silage. Furthermore, Rodrigues and Naehrer [73] reported that approximately 45% of 7049 samples of feed for livestock collected in America, Europe, and Asia contained ZEA with an average concentration of 233 μg/kg_DM_.

#### 3.2.3. FUM

The FUM content also decreased with increasing contamination of the samples, but the decrease was not statistically significant (Table 5, Figure 3). The average FUM content in all silage samples was 239.4 µg/kg_DM_. The lowest and highest values (194 and 296 µg/kg_DM_) were found in the MC variant.

The lowest and highest values (194 and 296 µg/kg_DM_) were found in the MC variant. The lowest and highest average values for maize silage published in the literature are 11.3 µg/kg_DM_ [70] and 3000 µg/kg_DM_ [77], respectively. In the Netherlands, Driehuis et al. [69] report a maximum value of 34,000 µg/kg_DM_ in maize silage. The most important factors favoring the secretion of fumonisins by Fusarium species are hot and dry periods, followed by conditions with increased humidity and damage by insects [69,77]. According to Rodrigues and Naehrer [73], fumonisins are the most common threat among mycotoxins for livestock, with an occurrence of 64% and an average concentration of 1965 μg/kg_DM_ in 7049 samples of livestock feed collected in America, Europe, and Asia.

According to Schmidt et al. [78], approximately 48.6% of 327 maize silages sampled in Brazil were contaminated with fumonisin B1; the average concentration was 369 μg/kg. Gonzalez-Pereyra et al. [56] reported that in their study, the content of fumonisin in maize silage ranged from 340 to 2490 μg/kg_DM_, with higher concentrations found in samples from the upper layer and sidewalls of the silage, which are usually more susceptible to degradation due to aerobic conditions and less intensive fermentation.

#### 3.2.4. AFL

**Figure 4:**
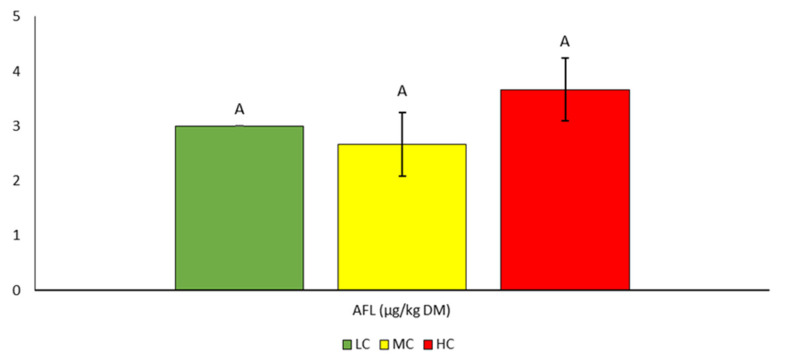
Contents of AFL mycotoxin in silage. Different letters indicate significant differences (*p* < 0.05) between variants (LC, MC, HC) in the specific parameter (content of individual mycotoxin).

The highest average aflatoxin content was found in the HC variant (3.7 µg/kg_DM_). However, the differences between the variants were not statistically significant (Table 5, Figure 4). The maximum value was 4.0 µg/kg. Limit values are specified, for example, in the European Commission Regulation No. 650/2010 with a maximum tolerated value of 5 µg AFLB1/kg.

In the literature, the average values of AFL content range from 3 µg/kg_DM_ [78] to 33 µg/kg_DM_ [79]. In particular, in the intercontinental research study by Rodrigues and Naehrer [73], aflatoxins were present in 33% of 7049 samples of feed for livestock at an average concentration of 63 μg/kg. And in the survey of Argentinian dairy farms that suspected problems with mycotoxins, about 30.2% of maize silage samples contained more than 20 μg/kg of aflatoxins, with a maximum concentration of 42.1 μg/kg.

AFL is one of the mycotoxins produced by *Aspergillus* spp. which are considered storage molds because they do not normally infect the crop before harvest. However, some species, such as *Aspergillus flavus*, can also infect plants in the field and produce aflatoxins during periods of high temperatures (>32 °C), high humidity (>80%), or drought stress [71,80].

The relationships among the individual mycotoxins were studied too. Table 6 shows a very strong negative dependence between DON and ZEA (R = −0.96) and ZEA and AFL (R = −0.78). This dependence indicates that higher contents of DON and AFL in silage can result in a lower content of ZEA, and to some extent also of FUM, which also has a negative (although weaker) correlation (R = −0.40) with the content of DON.

The correlation analysis further shows (Table A1) that the described relationships between the studied mycotoxins were likely to be caused by different requirements of molds for the environment [81]. In our study, a very strong positive correlation was observed between DON and pH; dry matter; and contents of NDF, ADF, CF, and lignin. On the other hand, a strong negative correlation was recorded between DON and the contents of lipids, sugar, LA, and AA. This character of correlations was to a lesser extent (moderately strong dependence) observed for AFL. By contrast, for ZEA and to a lesser extent for FUM, the correlations of the mentioned parameters were opposite.

Interactions between the mycotoxin content in the maize silage and the qualitative parameters were analyzed in detail using PCA (Appendix C). Three basic factors were identified (Table A4, Figure A2 and Figure A3) with Factor 1 and Factor 2 explaining more than 90% of the variability in the measured values (Figure A4). Factor 1 was labeled as the main factor as it explained 78.77% of the variability in the measured values. The factor correlated positively with the selected indicators of contaminated silage, but negatively with the production of biogas and its quality. It can therefore be assumed that the factor describes the level of maize silage contamination. Factor 2 also showed a negative correlation with the production of biogas and its quality, which was stronger than that of Factor 1. Considering that Factor 2 explained only 21.23% of the variability and had a demonstrably negative influence on biogas production, we assume that this factor represents a kind of mycotoxin.

### 3.3. Effect of Mycotoxins on the Production of Biogas and Methane

The production of biogas and methane ranged from 0.4305 (MC variant) to 0.6178 m^3^/kg_VS_ (LC variant) and from 0.2248 (MC variant) to 0.3792 m^3^/kg_VS_ (LC variant), respectively. The measured values are summarized in Table 7. The LC variant exhibited a statistically significantly higher biogas and methane yield than the MC and HC variants.

In particular, the methane concentration in the biogas showed no statistically significant differences between the respective variants. The final concentration of methane in the biogas ranged from 63%_vol_ to 64%_vol_ (Table 8).

**Figure 5:**
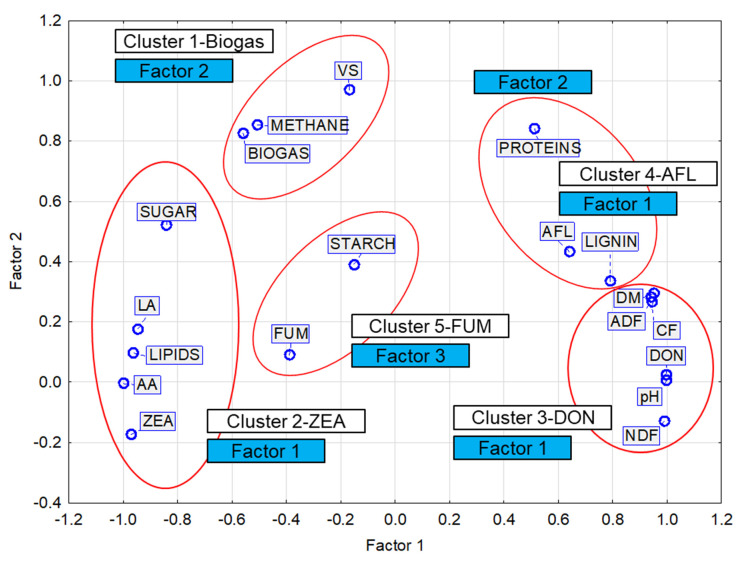
Factor loadings, Factor 1 vs. Factor 2. Extraction: principal components. Members of clusters (K-means clustering). VS—volatile solids; NDF—neutral detergent fiber; ADF—acid detergent fiber; CF—crude fiber. LA—lactic acid; AA—acetic acid; DON—deoxynivalenol; ZEA—zearalenone; FUM—fumonisin; AFL—aflatoxin.

**Figure A1:**
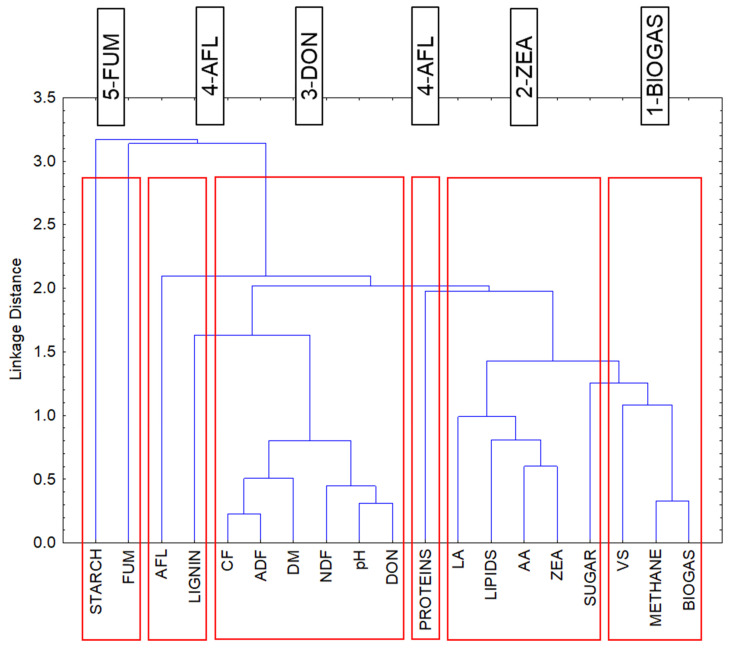
Results of cluster analysis: Tree diagram for 19 variables. Single linkage. Euclidean distances. K-means clustering of variables—members of clusters. NDF—neutral detergent fiber; ADF—acid detergent fiber; CF—crude fiber. LA—lactic acid; AA—acetic acid. DON—deoxynivalenol; ZEA—zearalenone; FUM—fumonisin; AFL—aflatoxin.

**Figure A2:**
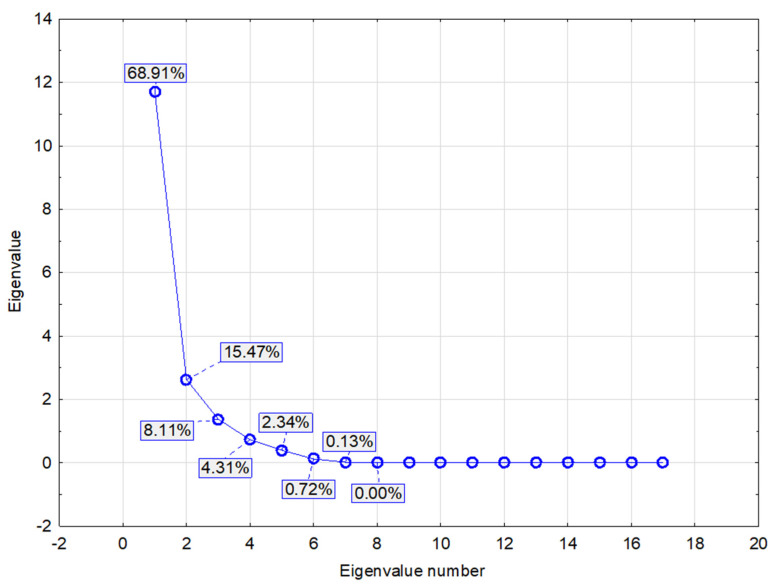
Plot of eigenvalues.

**Figure A3:**
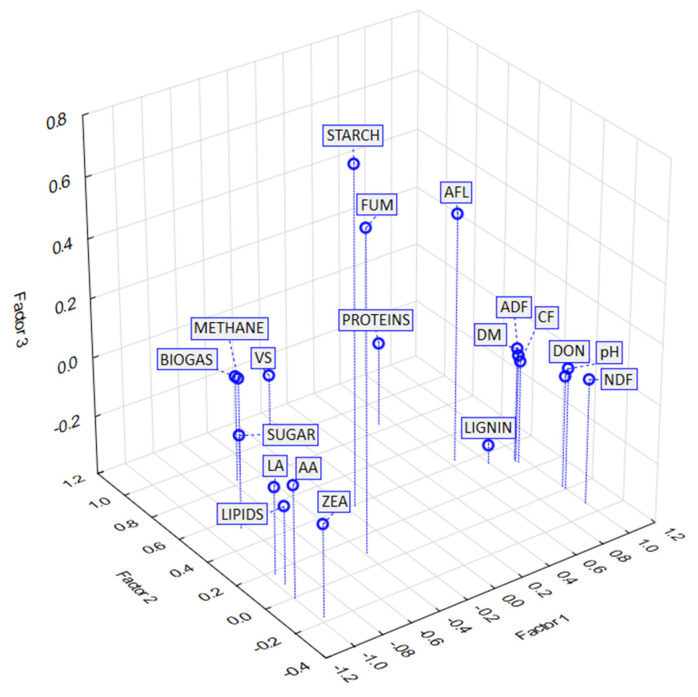
Factor analysis—factor loadings, Factor 1 vs. Factor 2 vs. Factor 3. Extraction: principal components. VS—volatile solids; NDF—neutral detergent fiber; ADF—acid detergent fiber; CF—crude fiber. LA—lactic acid; AA—acetic acid. DON—deoxynivalenol; ZEA—zearalenone; FUM—fumonisin; AFL—aflatoxin.

**Figure A4:**
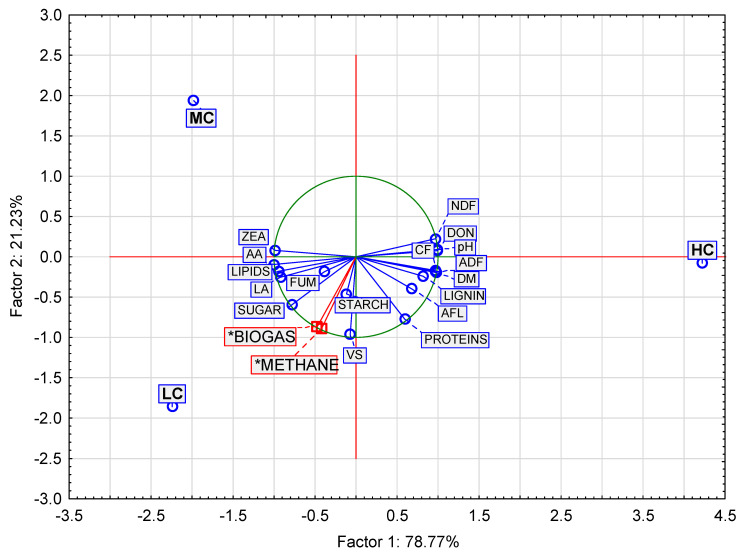
Projection of the variables on the factor plane (1 × 2) and projection of the cases (means) on the factor plane (1 × 2). Merged graphs. Total variance (Factor 1, Factor 2) values (%) of cases LC, MC, and HC are calculated from variable means. LC—low contamination; MC—medium contamination; HC—heavy contamination. VS—volatile solids; NDF—neutral detergent fiber; ADF—acid detergent fiber; CF—crude fiber. LA—lactic acid; AA—acetic acid. DON—deoxynivalenol; ZEA—zearalenone; FUM—fumonisin; AFL—aflatoxin. Symbol * indicates supplementary variables in biplot graph (PCA).

Correlation, cluster (Table A2), factor, and principal component analyses were carried out to classify the measured parameters and mycotoxin contents according to their similarity and the degree of their influence on the production of biogas and methane. The results of these analyses and their comparison are shown in Table 9 and Table A1, Table A2, Table A3, Table A4 and Table A5, as well as in Figure 5 and Figure A1, Figure A2, Figure A3 and Figure A4.

The resulting categorization into five clusters (Table 9) is consistent with the results of the factor analysis with three factors of which Factor 1 represents the members of three clusters: Clusters 2, 3 and 4. Cluster 2 includes variables with the negative factor loadings of Factor 1, while Clusters 3 and 4 have positive factor loadings (except proteins) (Table 9). The results of the correlation analysis (Table 9 and Table A1) show that none of the four mycotoxins has a significant correlation with the production of biogas or methane. As mentioned in the above subsection, Factor 1 explained the influence of the mycotoxin concentration in the silage, and Factor 2 explained the type of mycotoxin.

Although a moderately strong negative, statistically non-significant correlation was found between biogas and methane production and DON content (R = −0.54 and R = −0.48, respectively), this dependence can be considered secondary, as much stronger significant positive correlations were found between biogas and methane production and parameters belonging to Cluster 1 (VS) and Cluster 2 (sugars, lipids, and LA). In contrast, the DON content had a strong negative correlation with the members of Cluster 2 (Table A1). This result is consistent with the conclusions of Ferrara et al. [1], according to which the high content of mycotoxins had no influence on the overall production of biogas, especially methane. The same conclusions were reached by De Gelder et al. [24].

According to Merrettig-Bruns and Sayder [22], the fermentation of moldy maize silage as the only substrate led to a lower biogas yield compared to the fermentation of maize silage without molds. The authors also report that an extended analysis of subgroups revealed a significantly lower content of easily digestible carbohydrates in silages made from moldy maize.

The comparison of the LC and HC variants shows that the biogas and methane yield was primarily reduced by the statistically significant decrease in the contents of VS, sugars, lipids, LA, and AA (members of Cluster 1 and Cluster 2) and at the same time by an associated statistically significant increase in the contents of NDF, ADF, and CF (members of Cluster 3) in the HC variant. However. The content of ADF, CF, and lignin was lower in the MC variant (by 8–11%) than in the LC variant, and the content of NDF was higher (by 9%) than that in the LC variant. The comparison of the LC and MC samples also showed only a slight difference in the content of lipids, LA, and AC and in the value of LA/AA. This process was accompanied by increased values for DM, pH, and LA/AA and a reduced value for TA.

Obviously, there were technological errors in the silage treatment, which led to changes in the substrate composition, resulting in reduced production of LA and thus insufficient preservation of the silage and its devaluation. This is also indicated by the titratable acidity (TA) values, which decreased in statistically significant proportion to the degree of contamination (Table 3).

The primary factor that reduced the production of biogas and methane is therefore the aerobic degradation of the silage in correlation with the changes in its composition expressed by contents of VS, sugars, LA, and AA and their ratios. The ratios showed a moderately strong to strong positive correlation, increasing the production of both gases. In contrast, the biogas and methane yield decreased with increasing content of NDF, ADF, CF, and lignin. The secondary factor related to the changes in substrate composition was the development of undesirable microorganisms that further contributed to substrate degradation and mycotoxin production. According to McDonald et al. [82], yeasts can oxidize lactic acid and thus increase the pH of the silage, which favors the growth of other microorganisms.

The division of mycotoxins (DON, ZEA, FUM, AFL) into four clusters (Clusters 2, 3, 4, 5) could be related to the conditions under which microorganisms producing the respective toxins can develop successfully (Figure 5, Table A1).

1. Cluster 2—ZEA

ZEA has a strong positive correlation with the contents of lipids, sugar, LA, and AA and a negative correlation with the values of pH; DM; and the content of NDF, ADF, CF, and lignin.

2. Cluster 3—DON

DON has a strong positive correlation with the values of pH; DM; and the content of NDF, ADF, CF, and lignin and a negative correlation with the contents of lipids, sugar, LA, and AA.

3. Cluster 4—AFL

With respect to the mutual proximity of Cluster 3 (DON) and Cluster 4 (AFL) (Figure 5), the AFL content has a moderately strong positive correlation with the values of DM; pH; and lignin, NDF, ADF, and CF content and, similar to the DON content, a negative correlation with members of Cluster 2 (ZEA) (LA, AA, lipids).

4. Cluster 5—FUM

This cluster contains only FUM and starch. The contents of both FUM and starch show only a weak or slight dependence on the variables in the other clusters and also on each other. These two variables are the only ones represented by Factor 3 (Table 9). It can therefore be assumed that the levels of FUM and starch are related to parameters that were not considered in this study. This fact is illustrated in Figure A3.

Silages may contain a mixture of mycotoxins resulting from both pre-harvest contamination with *Fusarium* spp. and *Aspergillus* spp. [72,83] and post-harvest contamination by molds which commonly occur in silages, such as *Penicillium* spp. or *Aspergillus* spp. [84,85]. According to Zain [86], there are many common molds that do not produce mycotoxins. The presence of molds in silage does not have to always indicate the presence of mycotoxins, and the absence of molds does not indicate the absence of mycotoxins. Conditions for the growth of molds and for the development of mycotoxins do not have to be always the same [76]. Molds of *Fusarium* spp. for example can grow intensively at temperatures ranging from 25 to 30 °C without producing mycotoxins, while at temperatures below the freezing point, they produce large quantities of mycotoxins with minimal growth [76,87].

### 3.4. Content of Mycotoxins in Digestate

The comparison of the contents of mycotoxins DON, ZEA, FUM, and AFL in the silage and in the digestate (Table 10) shows that the mycotoxins were degraded during the anaerobic fermentation process, and their content was reduced by 27–100%. The only exception is the LC variant in which the concentration of DON increased by 27.8%. This can be explained by the fact that the sample contained the lowest initial concentration of DON which was not metabolized by the microorganisms. During the process of anaerobic digestion, the mass of the initial feedstock was reduced, which could have led to the higher concentration of DON in the digestate.

Similar results were also published by Ferrara et al. [1], who reported in their study that the degradation of mycotoxins was approx. 54% for aflatoxin B1 and 60% for fumonisins. De Gelder et al. [24] report that in batch tests with mycotoxins, aflatoxin B1, ochratoxin A, deoxynivalenol, zearalenone, and T-2 toxin were degraded by more than 90%. According to Richter et al. [88], the ensiling of DON-contaminated maize groats showed a significantly reduced DON content; the DON content was also reduced during the fermentation of whole-grain maize. These facts show that the use of mycotoxin-contaminated substrates for biogas production is a good way for their processing, although some studies conducted with mycotoxins in contaminated raw materials for biogas production indicated that the toxins are degraded at different rates during the anaerobic fermentation process [23,24,28]. However, further investigation of the effect of mycotoxins on the methanogenic archaea in the fermenter and the transfer of mycotoxin residues from the digestate to arable land and thus to crops is a necessary prerequisite.

The measured levels of mycotoxins in silage and the influence of these mycotoxins on the process of biogas production and possibly on the quality of digestate confirm that appropriate attention should be paid to the ensiling process. According to Rada and Vlková [89], the ensiling process requires quality control and strict organization. Compaction of the ensiled material (either maize biomass or biomass of forage plants) is elementary, and the air must be pressed out. The addition of silage inoculants (lactic acid bacteria) is also necessary. As far as the compaction of the silage and its covering are concerned, there are not too many alternative technologies that would differ significantly. With silage inoculants, however, you have the choice between a large number of homofermentative and heterofermentative lactic acid bacteria [90,91,92]. Homofermentative bacteria produce lactic acid, while heterofermentative bacteria are also able to produce acetic acid [90]. Heterofermentative inoculants in particular open the way for innovations in the ensiling process, as these bacteria can also produce enzymes, in addition to lactic and acetic acids, which contribute to better stability of the ensiling process and to the quality of the silage. According to Rada and Vlková [89], however, it is necessary to distinguish between the type of plant biomass to be ensiled (different inoculants for maize biomass and for crops difficult to ensile such as alfalfa) and the type of preparation (length of the chopped material). Therefore, the choice of an ensiling agent is an important part of the ensiling process, influencing not only the quality of the resulting silage, but also its durability.

## 4. Conclusions

The presence of mycotoxins in animal feed has an unfavorable effect on the performance and health of farm animals and can also endanger human health. This study has shown that the use of anaerobic digestion (AD) to produce biogas and methane is a promising method for utilizing mycotoxin-contaminated maize silage that cannot be used as feed for livestock. None of the four mycotoxins tested (deoxynivalenol, zearalenone, fumonisin, aflatoxin) in the silage had a significant effect on the production of biogas (methane), either alone or in combination with the others. This means that the alternative hypothesis H_1_ was rejected.

The observed decrease in biogas yield, which was proportional to the increasing contamination by molds, was primarily caused by the aerobic degradation of the silage in correlation with the changes in its nutrient composition, as reflected in the values of VS, sugar, lactic acid, and acetic acid contents and their ratios which exhibited a statistically significant moderately strong to strong positive correlation, thus increasing the production of the two gases, while an increase in the contents of NDF, ADF, CF, and lignin reduced the production of biogas and methane with increasing contamination. The secondary factor that correlated with the changes in substrate composition was the development of undesirable microorganisms that further contributed to substrate degradation and the production of mycotoxins. In addition to biogas, a by-product of the AD process is digestate, which can be of high agronomic value due to its high mineral nitrogen content and relatively stable organic matter. This study showed that the mycotoxins tested were degraded during the AD process and that their levels were reduced by 27–100%. Only in one case, in the variant with low mold contamination, the concentration of deoxynivalenol increased by 27.8%.

## Figures and Tables

**Table 1:** Contents of DM, pH, VS, proteins, lipids, and sugar in silage samples and results of Tukey’s HSD test.

Variant	DM	pH	VS	Proteins	Lipids	Sugar
	% ± SD	% ± SD	% ± SD	% ± SD	% ± SD	% ± SD
LC	36.55 ± 0.48 B	4.22 ± 0.02 C	94.38 ± 0.02 C	9.8 ± 0.01 B	2.13 ± 0.09 A	0.82 ± 0.03 A
MC	34.31 ± 0.07 C	4.51 ± 0.02 B	92.05 ± 0.06 A	8.42 ± 0.01 C	1.97 ± 0.07 A	0.48 ± 0.02 B
HC	47.92 ± 0.15 A	6.10 ± 0.01 A	93.17 ± 0.02 B	10.09 ± 0.12 A	1.52 ± 0.10 B	0.30 ± 0.02 C

Legend: LC—low contamination; MC—medium contamination; HC—heavy contamination. SD = standard deviation; HSD = post hoc Tukey’s test. Different letters indicate significant differences (*p* < 0.05) between respective variants in the specific parameter.

**Table 2:** Contents of starch, NDF, ADF, CF, and lignin in silage samples and results of Tukey’s HSD test.

Variant	Starch	NDF	ADF	CF	Lignin
	% ± SD	% ± SD	% ± SD	% ± SD	% ± SD
LC	28.38 ± 8.29 A	40.92 ± 0.51 C	24.7 ± 0.80 B	20.72 ± 0.04 B	3.93 ± 0.74 AB
MC	27.88 ± 7.76 A	44.56 ± 0.29 B	22.72 ± 0.34 B	19.14 ± 0.08 B	3.52 ± 0.15 B
HC	28.17 ± 8.05 A	53.89 ± 0.61 A	34.54 ± 1.79 A	29.48 ± 1.82 A	4.94 ± 0.03 A

Legend: LC—low contamination; MC—medium contamination; HC—heavy contamination. ±SD = standard deviation; HSD = post hoc Tukey’s test. Different letters indicate significant differences (*p* < 0.05) between respective variants in the specific parameter.

**Table 3:** Contents of lactic acid (LA) and acetic acid (AA), LA/AA, and titratable acidity of silage samples and results of Tukey’s HSD test.

Variant	Lactic Acid (LA)	Acetic Acid (AC)	LA/AA	Titratable Acidity (TA)
	% ± SD	% ± SD	% ± SD	% ± SD
LC	1.44 ± 0.05 A	1.42 ± 0.02 A	1.01 B	2063.00 ± 24.64 A
MC	1.17 ± 0.15 A	1.25 ± 0.01 B	0.94 B	991.671 ± 29.74 B
HC	0.65 ± 0.14 B	0.33 ± 0.01 C	1.99 A	803.332 ± 25.38 C

Legend: LC—low contamination; MC—medium contamination; HC—heavy contamination. ±SD = standard deviation; HSD = post hoc Tukey’s test. Different letters (A, B, and C) indicate significant differences (*p* < 0.05) between respective variants in the specific parameter.

**Table 4:** Descriptive statistics of DON and ZEA concentrations in maize silage.

Variant	DON (µg/kg_DM_)					ZEA (µg/kg_DM_)				
	Mean	±SD	Min	Max	HSD	Mean	±SD	Min	Max	HSD
LC	195.7	15.0	180.0	210.0	B	137.7	1.5	136.0	139.0	A
MC	251.0	20.1	230.0	270.0	B	138.7	8.0	131.0	147.0	A
HC	638.3	30.1	610.0	670.0	A	69.7	6.5	63.0	76.0	B

Legend: LC—low contamination; MC—medium contamination; HC—heavy contamination. ±SD = standard deviation; HSD = post hoc Tukey’s test. Different letters indicate significant differences (*p* < 0.05) between respective variants in the specific parameter.

**Table 5:** Descriptive statistics of FUM and AFL concentrations in maize silage.

Variant	FUM (µg/kg_DM_)					AFL (µg/kg_DM_)				
	Mean	±SD	Min	Max	HSD	Mean	±SD	Min	Max	HSD
LC	249.7	10.0	240.0	260.0	A	3.0	0.0	3.0	3.0	A
MC	243.7	51.1	194.0	296.0	A	2.7	0.6	2.0	3.0	A
HC	225.0	11.5	214.0	237.0	A	3.7	0.6	3.0	4.0	A

Legend: LC—low contamination; MC—medium contamination; HC—heavy contamination. ±SD = standard deviation; HSD = post hoc Tukey’s test. Different letters indicate significant differences (*p* < 0.05) between respective variants in the specific parameter.

**Table 6:** Summary of correlations between the measured variables.

Variant	Mean	±SD	DON	ZEA	FUM	AFL
DON	361.67	209.80	1.00	−0.96	−0.40	0.61
ZEA	115.33	34.65	−0.96	1.00	0.28	−0.78
FUM	239.44	28.88	−0.40	0.28	1.00	0.03
AFL	3.11	0.60	0.61	−0.78	0.03	1.00

Legend: DON—deoxynivalenol; ZEA—zearalenone; FUM—fumonisin; AFL—aflatoxin. Values shown in red indicate a strong correlation.

**Table 7:** Biogas and methane yield after 21 days of fermentation in three silage variants with different degrees of contamination.

Variant	Biogas (m^3^/kg_VS_)			Methane (m^3^/kg_VS_)		
	Mean	±SD	HSD	Mean	±SD	HSD
LC	0.6178	0.0032	A	0.3792	0.0119	A
MC	0.4305	0.0113	B	0.2248	0.0015	B
HC	0.4464	0.0204	B	0.2479	0.0135	B

Legend: LC—low contamination; MC—medium contamination; HC—heavy contamination. ±SD = standard deviation. HSD = post hoc Tukey’s test. Different letters indicate significant differences (*p* < 0.05) between respective variants.

**Table 8:** Concentration of methane in biogas after 21 days of fermentation in three silage variants with different degrees of contamination.

Variant	Methane Content (%_vol_)		
	Mean	±SD	HSD
LC	63	0.0	A
MC	64	0.5	A
HC	63.5	0.5	A

Legend: LC—low contamination; MC—medium contamination; HC—heavy contamination. ±SD = standard deviation. HSD = post hoc Tukey’s test. Different letters indicate significant differences (*p* < 0.05) between respective variants.

**Table 9:** Results of the correlation, cluster, factor, and principal component analyses.

Variable		Cluster Analysis	Correlation (R)	Factor Analysis	
		Euclidean Distance	R: Biogas vs. Variables ^1^	Factor	Factor Loading ^2^
Members of Cluster Number 1—BIOGAS					
1	BIOGAS	0.00	1.00	Factor 2	−0.81
2	METHANE	0.33	0.99	Factor 2	−0.84
3	VS	1.26	0.90	Factor 2	−0.97
Members of Cluster Number 2—ZEA					
1	ZEA	3.11	0.39	Factor 1	−0.97
2	LIPIDS	2.43	0.63	Factor 1	−0.96
3	SUGAR	1.25	0.90	Factor 1	−0.85
4	LA	2.26	0.68	Factor 1	−0.95
5	AA	2.68	0.55	Factor 1	−1.00
Members of Cluster Number 3—DON					
1	DON	4.96	−0.54	Factor 1	1.00
2	pH	4.98	−0.55	Factor 1	1.00
3	DM	4.53	−0.28	Factor 1	0.94
4	NDF	5.15	−0.66	Factor 1	0.99
5	ADF	4.56	−0.30	Factor 1	0.93
6	CF	4.58	−0.31	Factor 1	0.94
Members of Cluster Number 4—AFL					
1	AFL	4.00	0.00	Factor 1	0.63
2	LIGNIN	4.30	−0.15	Factor 1	0.78
3	PROTEINS	3.07	0.41	Factor 2	−0.86
Members of Cluster Number 5—FUM					
1	FUM	3.41	0.27	Factor 3	0.67
2	STARCH	3.20	0.11	Factor 3	0.76

Legend: ^1^ Correlation between biogas yield and other parameters. Marked correlations are significant at *p* < 0.05. ^2^ Highlight factor (red-marked) loadings ≥ 0.70. NDF—neutral detergent fiber; ADF—acid detergent fiber; CF—crude fiber. LA—lactic acid; AA—acetic acid; DON—deoxynivalenol; ZEA—zearalenone; FUM—fumonisin; AFL—aflatoxin.

**Table 10:** Content of mycotoxins (DON, ZEA, FUM, AFL) in the three variants (LC, MC, HC) of silage before the AD process and in the digestate after the AD process and their difference.

Variant	FUM (µg/kg_DM_)					AFL (µg/kg_DM_)					Difference	
	Mean	± SD	Min	Max	HSD	Mean	± SD	Min	Max	HSD	Mean (µg/kg_DM_)	Mean (%)
DON-LC	195.7	15.0	180.0	210.0	B	250.0	20.0	230.0	270.0	A	54.3	27.8
DON-MC	251.0	20.1	230.0	270.0	B	183.3	15.3	170.0	200.0	B	−67.7	−27.0
DON-HC	638.3	30.1	610.0	670.0	A	150.0	30.0	120.0	180.0	B	−488.3	−76.6
ZEA-LC	137.7	1.5	136.0	139.0	A	26.7	2.1	25.0	29.0	B	−111.0	−80.6
ZEA-MC	138.7	8.0	131.0	147.0	A	29.3	4.5	25.0	34.0	AB	−109.3	−78.7
ZEA-HC	69.7	6.5	63.0	76.0	B	36.7	3.5	33.0	40.0	A	−42.8	−61.4
FUM-LC	249.7	10.0	240.0	260.0	A	82.7	6.0	77.0	89.0	C	−167.0	−66.9
FUM-MC	243.7	51.1	194.0	296.0	A	114.7	3.1	112.0	118.0	A	−129.0	−51.7
FUM-HC	225.0	11.5	214.0	237.0	A	98.0	5.6	92.0	103.0	B	−107.5	−47.7
AFL-LC	3.0	0.0	3.0	3.0	A	0.0	0.0	0.0	0.0	-	−3.0	−100.0
AFL-MC	2.7	0.6	2.0	3.0	A	0.0	0.0	0.0	0.0	-	−2.7	−100.0
AFL-HC	3.7	0.6	3.0	4.0	A	0.0	0.0	0.0	0.0	-	−3.7	−100.0

Legend: LC—low contamination; MC—medium contamination; HC—heavy contamination. DON—deoxynivalenol; ZEA—zearalenone; FUM—fumonisin; AFL—aflatoxin. ±SD = standard deviation. HSD = post hoc Tukey’s test. Different letters indicate significant differences (*p* < 0.05) between the respective variants.

## Data Availability

The data that support the findings of this study are available on request from the corresponding author (J.E.).

## Appendix A. Correlation Analysis

**Table A1:** Correlation matrix. Red-marked correlation coefficients are significant at *p* < 0.05.

Variable	BIOGAS	METHANE	DON	ZEA	FUM	AFL	pH	DM	VS	NDF	ADF	CF	LIGNIN	STARCH	PROTEINS	LIPIDS	SUGAR	LA	AA
BIOGAS	1.00	0.99	−0.54	0.39	0.27	0.00	−0.55	−0.28	0.90	−0.66	−0.30	−0.31	−0.15	0.36	0.41	0.63	0.90	0.68	0.55
METHANE	0.99	1.00	−0.48	0.35	0.21	0.00	−0.50	−0.22	0.93	−0.61	−0.23	−0.25	−0.13	0.37	0.47	0.57	0.87	0.65	0.50
DON	−0.54	−0.48	1.00	−0.96	−0.40	0.61	0.99	0.96	−0.14	0.99	0.96	0.96	0.77	−0.14	0.54	−0.96	−0.82	−0.93	−0.99
ZEA	0.39	0.35	−0.96	1.00	0.28	−0.78	−0.98	−0.98	0.01	−0.94	−0.95	−0.94	−0.80	0.05	−0.63	0.90	0.74	0.90	0.98
FUM	0.27	0.21	−0.40	0.28	1.00	0.03	−0.38	−0.36	0.08	−0.39	−0.33	−0.32	−0.47	0.29	−0.15	0.37	0.38	0.17	0.38
AFL	0.00	0.00	0.61	−0.78	0.03	1.00	0.66	0.72	0.21	0.57	0.63	0.63	0.60	0.35	0.59	−0.58	−0.36	−0.55	−0.66
pH	−0.55	−0.50	0.99	−0.98	−0.38	0.66	1.00	0.95	−0.17	0.99	0.94	0.94	0.79	−0.15	0.51	−0.95	−0.84	−0.94	−1.00
DM	−0.28	−0.22	0.96	−0.98	−0.36	0.72	0.95	1.00	0.13	0.91	0.98	0.98	0.83	−0.04	0.74	−0.89	−0.65	−0.84	−0.96
VS	0.90	0.93	−0.14	0.01	0.08	0.21	−0.17	0.13	1.00	−0.29	0.13	0.12	0.22	0.28	0.76	0.27	0.67	0.34	0.17
NDF	−0.66	−0.61	0.99	−0.94	−0.39	0.57	0.99	0.91	−0.29	1.00	0.90	0.91	0.71	−0.17	0.40	−0.97	−0.90	−0.96	−0.99
ADF	−0.30	−0.23	0.96	−0.95	−0.33	0.63	0.94	0.98	0.13	0.90	1.00	1.00	0.80	−0.05	0.75	−0.90	−0.64	−0.85	−0.94
CF	−0.31	−0.25	0.96	−0.94	−0.32	0.63	0.94	0.98	0.12	0.91	1.00	1.00	0.83	−0.08	0.74	−0.89	−0.64	−0.87	−0.94
LIGNIN	−0.15	−0.13	0.77	−0.80	−0.47	0.60	0.79	0.83	0.22	0.71	0.80	0.83	1.00	−0.27	0.70	−0.63	−0.44	−0.68	−0.78
STARCH	0.36	0.37	−0.14	0.05	0.29	0.35	−0.15	−0.04	0.28	−0.17	−0.05	−0.08	−0.27	1.00	0.15	0.00	0.24	0.23	0.14
PROTEINS	0.41	0.47	0.54	−0.63	−0.15	0.59	0.51	0.74	0.76	0.40	0.75	0.74	0.70	0.15	1.00	−0.41	0.02	−0.35	−0.51
LIPIDS	0.63	0.57	−0.96	0.90	0.37	−0.58	−0.95	−0.89	0.27	−0.97	−0.90	−0.89	−0.63	0.00	−0.41	1.00	0.87	0.92	0.96
SUGAR	0.90	0.87	−0.82	0.74	0.38	−0.36	−0.84	−0.65	0.67	−0.90	−0.64	−0.64	−0.44	0.24	0.02	0.87	1.00	0.87	0.84
LA	0.68	0.65	−0.93	0.90	0.17	−0.55	−0.94	−0.84	0.34	−0.96	−0.85	−0.87	−0.68	0.23	−0.35	0.92	0.87	1.00	0.94
AA	0.55	0.50	−0.99	0.98	0.38	−0.66	−1.00	−0.96	0.17	−0.99	−0.94	−0.94	−0.78	0.14	−0.51	0.96	0.84	0.94	1.00

## Appendix B. Cluster Analysis

**Table A2:** Euclidean distances.

Variable	BIOGAS	METHANE	DON	ZEA	FUM	AFL	pH	DM	VS	NDF	ADF	CF	LIGNIN	STARCH	PROTEINS	LIPIDS	SUGAR	LA	AA
BIOGAS	0.00	0.33	4.96	3.11	3.41	4.00	4.98	4.53	1.26	5.15	4.56	4.58	4.30	3.20	3.07	2.43	1.25	2.26	2.68
METHANE	0.33	0.00	4.86	3.23	3.56	4.00	4.90	4.42	1.08	5.07	4.43	4.47	4.24	3.17	2.92	2.62	1.45	2.36	2.83
DON	4.96	4.86	0.00	5.61	4.74	2.50	0.31	0.80	4.26	0.46	0.81	0.80	1.90	4.28	2.70	5.61	5.40	5.56	5.65
ZEA	3.11	3.23	5.61	0.00	3.39	5.33	5.63	5.63	3.97	5.57	5.58	5.58	5.36	3.89	5.10	1.24	2.05	1.29	0.60
FUM	3.41	3.56	4.74	3.39	0.00	3.93	4.70	4.66	3.85	4.71	4.62	4.60	4.85	3.37	4.30	3.18	3.16	3.65	3.14
AFL	4.00	4.00	2.50	5.33	3.93	0.00	2.33	2.10	3.56	2.63	2.42	2.43	2.53	3.23	2.55	5.02	4.66	4.98	5.15
pH	4.98	4.90	0.31	5.63	4.70	2.33	0.00	0.85	4.32	0.45	1.00	0.97	1.84	4.28	2.79	5.59	5.43	5.58	5.66
DM	4.53	4.42	0.80	5.63	4.66	2.10	0.85	0.00	3.73	1.22	0.51	0.57	1.63	4.08	2.03	5.49	5.14	5.43	5.59
VS	1.26	1.08	4.26	3.97	3.85	3.56	4.32	3.73	0.00	4.55	3.72	3.75	3.52	3.40	1.97	3.42	2.32	3.26	3.65
NDF	5.15	5.07	0.46	5.57	4.71	2.63	0.45	1.22	4.55	0.00	1.25	1.23	2.16	4.33	3.09	5.62	5.52	5.60	5.64
ADF	4.56	4.43	0.81	5.58	4.62	2.42	1.00	0.51	3.72	1.25	0.00	0.23	1.78	4.11	2.02	5.51	5.12	5.44	5.57
CF	4.58	4.47	0.80	5.58	4.60	2.43	0.97	0.57	3.75	1.23	0.23	0.00	1.64	4.16	2.05	5.50	5.12	5.47	5.57
LIGNIN	4.30	4.24	1.90	5.36	4.85	2.53	1.84	1.63	3.52	2.16	1.78	1.64	0.00	4.50	2.17	5.11	4.80	5.19	5.33
STARCH	3.20	3.17	4.28	3.89	3.37	3.23	4.28	4.08	3.40	4.33	4.11	4.16	4.50	0.00	3.70	4.00	3.49	3.51	3.72
PROTEINS	3.07	2.92	2.70	5.10	4.30	2.55	2.79	2.03	1.97	3.09	2.02	2.05	2.17	3.70	0.00	4.74	3.95	4.64	4.92
LIPIDS	2.43	2.62	5.61	1.24	3.18	5.02	5.59	5.49	3.42	5.62	5.51	5.50	5.11	4.00	4.74	0.00	1.43	1.15	0.81
SUGAR	1.25	1.45	5.40	2.05	3.16	4.66	5.43	5.14	2.32	5.52	5.12	5.12	4.80	3.49	3.95	1.43	0.00	1.45	1.59
LA	2.26	2.36	5.56	1.29	3.65	4.98	5.58	5.43	3.26	5.60	5.44	5.47	5.19	3.51	4.64	1.15	1.45	0.00	0.99
AA	2.68	2.83	5.65	0.60	3.14	5.15	5.66	5.59	3.65	5.64	5.57	5.57	5.33	3.72	4.92	0.81	1.59	0.99	0.00

**Table A3:** Results of cluster analysis: K-means clustering of variables. Members of clusters and distances from respective cluster center.

Cluster 1 (Biogas)			Cluster 2 (ZEA)			Cluster 3 (DON)			Cluster 4 (AFL)			Cluster 5 (FUM)		
Variable		Distance	Variable		Distance	Variable		Distance	Variable		Distance	Variable		Distance
1	BIOGAS	0.165876	1	ZEA	0.308224	1	DON	0.099882	1	AFL	4.00	1	FUM	0.561778
2	METHANE	0.109718	2	LIPIDS	0.215193	2	pH	0.151874	2	LIGNIN	4.30	2	STARCH	0.561778
3	VS	0.258680	3	SUGAR	0.404457	3	DM	0.178573	3	PROTEINS	3.07			
			4	LA	0.240395	4	NDF	0.245168						
				AA	0.151888	5	ADF	0.182380						
						6	CF	0.180448						

## Appendix C. Factor Analysis

**Table A4:** Factor loadings. Extraction: principal components. Highlighted (red-marked) factor loadings > 0.70.

Variable	Factor 1	Factor 2	Factor 3
BIOGAS	−0.58	−0.81	0.00
METHANE	−0.53	−0.84	−0.03
DON	1.00	−0.05	−0.02
ZEA	−0.97	0.19	−0.10
FUM	−0.39	−0.06	0.67
AFL	0.63	−0.42	0.46
pH	1.00	−0.03	0.01
DM	0.94	−0.32	0.00
VS	−0.19	−0.97	−0.12
NDF	0.99	0.11	0.02
ADF	0.93	−0.31	−0.02
CF	0.94	−0.29	−0.04
LIGNIN	0.78	−0.36	−0.32
STARCH	−0.16	−0.35	0.76
PROTEINS	0.49	−0.86	−0.08
LIPIDS	−0.96	−0.08	−0.13
SUGAR	−0.85	−0.51	−0.06
LA	−0.95	−0.16	−0.10
AA	−1.00	0.03	−0.02

**Table A5:** Eigenvalues. Extraction: principal components.

Value Number	Eigenvalue	% Total	Cumulative Eigenvalue	Cumulative %
1	11.71	68.91	11.71	68.91
2	2.63	15.47	14.34	84.38
3	1.38	8.11	15.72	92.49
